# Enhancement of Bacterial Anti-Adhesion Properties on Robust PDMS Micro-Structure Using a Simple Flame Treatment Method

**DOI:** 10.3390/nano12030557

**Published:** 2022-02-06

**Authors:** Nongluck Houngkamhang, Ploymanee Chaisawat, Waisaree Joksathit, Sutichai Samart, Sutee Chutipaijit, Suphichaya Radomyos, Pawasuth Saengdee, Nithi Atthi

**Affiliations:** 1 College of Materials Innovation and Technology, King Mongkut’s Institute of Technology Ladkrabang (KMITL), Bangkok 10520, Thailand; nongluck.ho@kmitl.ac.th (N.H.); 61110108@kmitl.ac.th (P.C.); 61110073@kmitl.ac.th (W.J.); sutichai.sa@kmitl.ac.th (S.S.); sutee.ch@kmitl.ac.th (S.C.); 2 Thai Microelectronics Center (TMEC), National Electronics and Computer Technology Center (NECTEC), Chachoengsao 24000, Thailand; suphichaya.rad@ncr.nstda.or.th (S.R.); pawasuth.sae@nectec.or.th (P.S.)

**Keywords:** antifouling, bacterial anti-adhesion, *Escherichia coli*, flame treatment, flower petal nano-structure, PDMS, robust micro-structure, soft lithography, superhydrophobic

## Abstract

Biofilm-associated infections caused by an accumulation of micro-organisms and pathogens significantly impact the environment, health risks, and the global economy. Currently, a non-biocide-releasing superhydrophobic surface is a potential solution for antibacterial purposes. This research demonstrated a well-designed robust polydimethylsiloxane (PDMS) micro-structure and a flame treatment process with improved hydrophobicity and bacterial anti-adhesion properties. After the flame treatment at 700 ± 20 °C for 15 s, unique flower-petal re-entrant nano-structures were formed on pillars (PIL-F, width: 1.87 ± 0.30 μm, height: 7.76 ± 0.13 μm, aspect ratio (A.R.): 4.14) and circular rings with eight stripe supporters (C-RESS-F, width: 0.50 ± 0.04 μm, height: 3.55 ± 0.11 μm, A.R.: 7.10) PDMS micro-patterns. The water contact angle (WCA) and ethylene glycol contact angle (EGCA) of flame-treated flat-PDMS (FLT-F), PIL–F, and C–RESS-F patterns were (133.9 ± 3.8°, 128.6 ± 5.3°), (156.1 ± 1.5°, 151.5 ± 2.1°), and (146.3 ± 3.5°, 150.7 ± 1.8°), respectively. The *Escherichia coli* adhesion on the C-RESS-F micro-pattern with hydrophobicity and superoleophobicity was 42.6%, 31.8%, and 2.9% less than FLT-F, PIL-F, and Teflon surfaces. Therefore, the flame-treated C-RESS-F pattern is one of the promising bacterial anti-adhesion micro-structures in practical utilization for various applications.

## 1. Introduction

**Figure 1:**
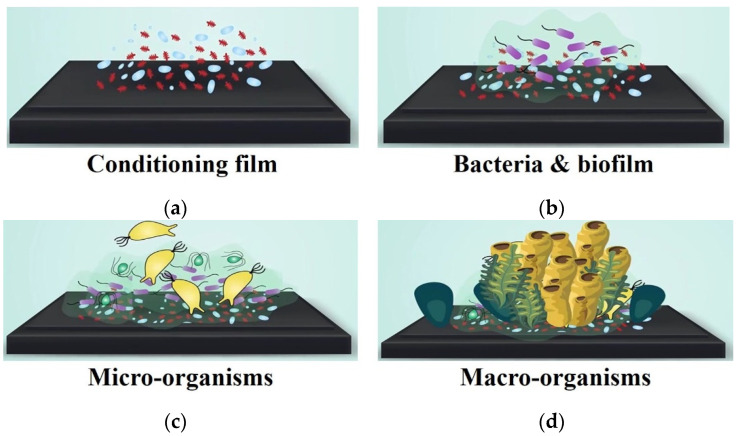
Biofilm formation involves four main stages of colonization. (**a**) The initial attachment of protein generates biofilm on the material surface; (**b**) bacteria and other bio-substances are attached to the biofilm; (**c**) bio-substances are continuously accumulated to create colonization of micro-organisms; (**d**) accumulation of macro-organisms.

Biofilm-associated infections are a globally important issue that poses significant severe health risks that are caused by the accumulation of microorganisms (bacteria, archaea, fungi, algae, protozoa, and viruses) and macromolecules (primarily proteins) on material surfaces [1,2]. Biofilms can cause the spread of various infectious diseases, including COVID-19, in public spaces and could lead to more than 5.73 million deaths worldwide in 2022 due to the COVID-19 pandemic [3]. The subsequent biofilm formation and the accumulation of contamination on wetted surfaces pose challenges in various applications such as health care, marine infrastructures, industry, engineering components, and public transportation systems [4]. This leads to a significant impact on the environment, health risks, society, and economics. For antibacterial solutions in medical devices and medical environments, such as a surgery rooms and ward areas, biocide-based antibacterial metallic ions such as silver (Ag), Titanium dioxide (TiO_2_), and zinc oxide (ZnO) are commonly used [5,6]. However, this oldest antibacterial method is not environmentally friendly and increases bacterial resistance and resistance to clinically important antibiotics [7]. Biofilm formation involves four main stages of colonization, as shown in Figure 1a–d. First, the initial attachment of protein generates biofilm on the material surface. Then, the bacteria and other bio-substances are attached to the biofilm. The bio-substances are continuously accumulated to create colonization of micro-organisms and macro-organisms. To prevent bacterial adhesion, the biofilm formation at the initial stage of the colonization mechanism should be terminated. Therefore, a non-biocide-release superhydrophobic surface is a new potential solution for reducing pathogen spread and other antibacterial purposes [8,9,10,11,12]. Low surface energy (γ_s_) material such as Teflon (γ_s_ ≈ 19.6 mN m^−1^) is currently used to reduce bacterial adhesion by reducing the adhesion force between bacteria and a solid surface, which can prevent biofilm formation on the surface [13].

**Figure 2:**
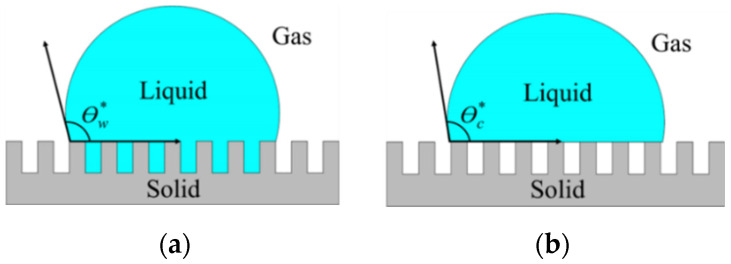
A liquid droplet resting on a solid surface with micro-/nano-structures: (**a**) the liquid is in intimate contact with the solid asperities of the rough surface so that the droplet is in the Wenzel state; (**b**) the liquid rests on top of the asperities of the rough surface so that the droplet is in the Cassie–Baxter state.

Instead of using expensive materials such as Teflon, a low-cost non-biocide-based superhydrophobic surface is one of the promising technologies for antifouling purposes. The levels of hydrophobicity and surface wettability can be determined by the water contact angle (WCA) [14,15,16]. If the contact angle of the water (surface tension, γ_lv_ = 72.1 mN m^−1^) is greater than 150° and has a water droplet sliding angle smaller than 10°, it is considered superhydrophobic. A water droplet can easily roll off the surface and carries off organisms, pathogens, inorganic macromolecules, and other contaminants that have a low adhesive force on the superhydrophobic surface [16]. Most engineering superhydrophobic surfaces are inspired by various natural animals and plants, such as lotus leaves, red rose petals, butterfly wings, Salvinia leaves, and shark skin, which show exceptional wetting behavior with superhydrophobicity [17,18,19,20,21,22]. The effect of surface roughness on the apparent contact angle of liquid droplets can be explained by the Wenzel and Cassie–Baxter model [23,24,25]. The liquid wets the surface in the Wenzel state and fills all voids on the rough surface, as shown in Figure 2a. In the Cassie–Baxter state, microscopic air pockets fill in the space between the rough micro- or nano-structures and the liquid droplets generate the ultimate liquid-repellent surface, as shown in Figure 2b [16].

It is well-known that a superhydrophobic surface that mimics nature-inspired features can be fabricated by creating a rough surface consisting of a micro- or nano-structure on a low-surface-energy material [15,16]. Polydimethylsiloxane (PDMS, γ_s_: ~12–16 mJ m^−2^), which is curable silicone-based material, is commonly used to fabricate superhydrophobic surfaces. PDMS also shows good thermal and oxidative stability, non-toxicity, good biocompatibility, and low cost [26]. Typically, the flat PDMS surface shows hydrophobic properties with a WCA of 107°–110°. Therefore, the fabrication of a rough texture with micro-/nano-structures such as micro-pillar (PIL) and biomimetic shark skin features using soft lithography and large-area patterning processes makes the superhydrophobic PDMS surface [27,28]. However, PDMS surfaces with superhydrophobic properties still have some drawbacks that restrict their practical application in certain circumstances. Firstly, the liquid-repellent property is drastically reduced for low-surface-tension liquids such as oil, grease, ethylene glycol (EG, γ_lv_ = 47.3 mN m^−1^), decane (γ_lv_ = 23.8 mN m^−1^), and octane (γ_lv_ = 21.6 mN m^−1^). Secondly, superhydrophobicity and antibacterial properties are dramatically diminished for sticky pollutants. Thirdly, PDMS with a low Young’s modulus (~2.0 MPa) also includes deformation, merging, and collapsing structures, resulting in decreased hydrophobicity [29]. Therefore, well-designed robust PDMS micro-structures with superamphiphobic properties (water- and oil-repellent) are required to prevent the pattern from collapsing and maintain its antibacterial/antifouling properties for practical utilization.

It has been reported that circular rings with an eight stripe supporters (C-RESS) pattern are one of the promising robust antifouling micro-structures in practical utilization, such as marine antifouling in the seawater environment [30]. However, the marine antifouling properties of the PDMS-C-RESS micro-structure have tended to degrade after immersion in a seawater environment for five months [30]. This might be due to the degradation of the hydrophobicity of the PDMS-C-RESS micro-structure by sticky pollutants and low-surface-energy liquids in the seawater. Therefore, a superamphiphobic surface is necessary to overcome the limitation of the robust PDMS surface with superhydrophobic properties for practical utilization. To obtain superamphiphobic properties, re-entrant micro-structure and nano-roughness are required. The re-entrant micro-structure (surface having concave topographic features) can repel liquids with a wide range of surface tensions [31,32,33]. However, the inverted trapezoidal shape of the re-entrant micro-/nano-structures limits its mold release ability in soft lithography and roll-to-roll (R2R) processes for mass production [34]. Moreover, nano-scale features are difficult to replicate on the PDMS surface to create the high surface roughness due to the limitation of resolution of the conventional lithography process to generate the nano-structure on a master template. Therefore, the hierarchy of micro-/nano-structures with additional flame treatment [35,36,37] is one of the promising methods to create the re-entrant micro-/nanostructures with nano-roughness on the PDMS surface due to its simplicity and cost-effectiveness. Using a simple flame treatment process, we have demonstrated the formation of a re-entrant micro-structure on robust PDMS feature with superhydrophobic (water repellent) and superoleophobic properties (oil repellent) [38]. The mechanism of forming the flower petal nano-structure that creates the re-entrant micro-structure and lower surface energy of the PDMS patterns was explained [38]. The effectiveness of the robust C-RESS patterns in suppressing biofouling in the marine environment was demonstrated in our previous study [30]. However, the geometric dimensions, surface roughness, and the re-entrant micro-structure of the C-RESS-F pattern affecting the bacterial anti-adhesion remain unrevealed.

In this research, the effects of the micro-structure geometries of untreated PIL and C-RESS patterns and flame-treated PIL-F and C-RESS-F patterns on the PDMS hierarchical surface roughness, hydrophobicity, and bacterial anti-adhesion properties were investigated.

## 2. Materials and Methods

### 2.1. Silicon Master Mold Fabrication

Two different PDMS micro-structures, including square-shaped pillar (PIL) patterns arranged in a square array and circular rings with eight stripe supporters (C-RESS) patterns arranged in a hexagonal array, were designed. Based on Wenzel’s model, maximum WCA can be obtained by maximizing the roughness factor (r) by reducing pattern width (a) and pattern spacing (b) down to nanometer scale and by increasing pillar height (h) [23,24,25]. This means a maximum value of packing factor (P = a/b) and a maximum aspect ratio value (A.R. = h/a) are required to produce a superhydrophobic surface. In this experiment, the resolution of the lithographic exposure tool was 0.5 µm. Due to the Young’s modulus of PDMS being relatively low (~2.0 MPa), the fabrication of high A.R. structures is very challenging. Based on this limitation, the PIL pattern was designed with a width (a) of 2.0 μm, a pattern spacing (b) of 2.0 μm, and a pattern height (h) of 5.0 μm. In contrast, a robust C-RESS pattern was designed with a width (a) of 0.5 μm, a pattern spacing (b) of 0.5 μm, and a pattern height (h) of 2.5 μm. The design aspect ratios (A.R.s) of PIL and C-RESS micro-structures were 2.5 and 5.0, respectively.

**Figure 3:**
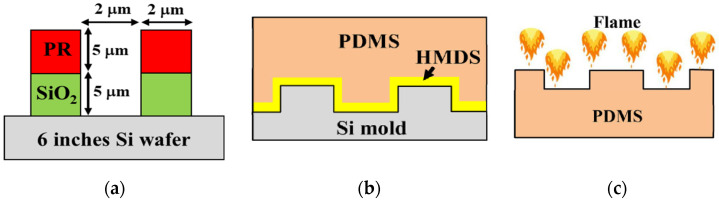
Cross-sectional schematic diagram (not to scale) of (**a**) PR/SiO_2_/Si patterning by conventional lithography and plasma etching process; (**b**) HMDS priming and PDMS casting on silicon master mold by soft lithography process; (**c**) simple flame treatment on PDMS.

To fabricate a silicon master mold (Si mold), a six-inch Si wafer was cleaned by a standard cleaning (SC-1) process. Then, a 5.0-µm-thick silicon dioxide (SiO_2_) hard mask layer was deposited onto the Si wafer using the plasma-enhanced chemical vapor deposition (PECVD) method (AMAT model P5000, Applied Materials Inc., Santa Clara, CA, USA). The PIL and C-RESS patterns were fabricated on a 5.0-µm-thick photoresist (PR) film by a conventional photolithography process using a stepper tool (Nikon stepper, NSR2005i8A, Nikon Corporation, Tokyo, Japan). Later, PIL and C-RESS patterns were transferred onto the SiO_2_ hard mask layer by a dry plasma reactive ion etching (RIE, AMAT model P5000, Applied Materials Inc., Santa Clara, CA, USA) process (CF_4_/CHF_3_/Ar gas flow rate: 10/50/100 sccm; pressure: 150 mTorr; RF power: 800 W/20 min). Then, the 5.0-µm-high PIL pattern and the 2.5-µm-high C-RESS patterns were etched into Si wafers by deep reactive ion etching (DRIE, Plasmalab system 100, Oxford instruments, Oxford, UK) using Bosch processes of 7 and 4 cycles, respectively, as shown in Figure 3a. One ion etching cycle included a deposition step (C_4_F_8_/SF_6_ gas flow rate: 200/5 sccm; ICP/RIE power: 2000/10 W; deposited time: 8 s) and an etching step (SF_6_ gas flow rate: 400 sccm; ICP/RIE power: 200/25 W; etching time: 5 s). Then, the remaining photoresist film was stripped by Piranha acid solution. The wet etching process removed the SiO_2_ hard mask to obtain the Si master molds [38].

### 2.2. Soft Lithography Process for PDMS Replication

Priming the Si molds in a hexamethyldisilazane (HMDS, Merck group, Darmstadt, Germany) mold releasing agent is required to prevent mold damage from hard PDMS residue in a poor demolding process. In this experiment, the Si molds were primed in the desiccator chamber with HMDS vapor for 48 h [39]. The PDMS (Sylgard 184, Dow Inc., Dow Way Midland, MI, USA) with the ratio between resin (part A) and curing agent (part B) of 10:1 wt% was poured onto the Si mold and cured at 75 °C for 120 min. The Sylgard-184 PDMS was released from the Si mold, as shown in Figure 3b. The PDMS flat surface (FLT) and Teflon flat surface were also fabricated as control samples.

### 2.3. Modification of PDMS Surface by the Flame Treatment Process

**Figure 4:**
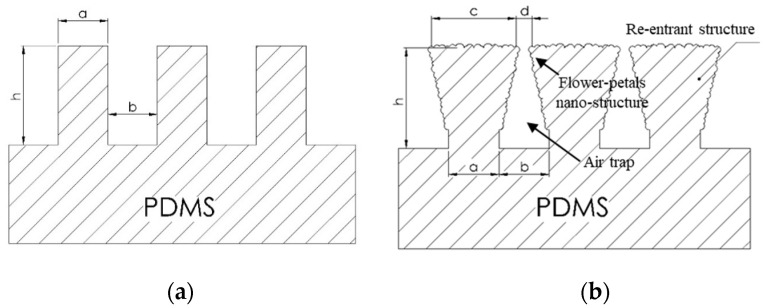
Schematic diagram of the formation of the re-entrance micro-structure by using a simple flame treatment process: (**a**) PDMS micro-structure before flame treatment where “a” is a designed pattern width, “b” is a designed pattern spacing, and “h” is a pattern height; (**b**) PDMS micro-structure with flower petal nano-structure after flame treatment where “a” is a designed pattern width, “b” is a designed pattern spacing, “c” is a pattern width of the top surface after flame treatment process, “d” is a pattern spacing of the top surface after flame treatment process, and “h” is a pattern height.

The flower petal nano-structure on the PDMS surface was developed by surface modification using a simple flame treatment process, as shown in Figure 3c and Figure 4a,b [35,36,37,38]. This flower petal nano-structure behaves like a re-entrant micro-/nano-structure, exhibiting superhydrophobic and superoleophobic surfaces in the Cassie–Baxter state. An amount of 2.64 g of bulk paper wipe was used as the flame source in this process. An amount of 11.26 g of ethanol (C_2_H_5_OH, Merck group, Darmstadt, Germany) was used as a burner to produce a large flame size with good flame stability. Then, 13.90 g of soaked paper wipe was burned for 1 min to remove its contamination and stabilize the laminar flame profile with the temperature of the post-reaction zone at 700 ± 20 °C. The flame-to-surface distance was kept at 20 mm. Three sets of the PDMS samples were exposed to the post-reaction zone of the flame by moving back and forth along their 2.0 cm width from one end to the other within 1 s for eight times, resulting in a flame contact time of 15 s [38]. Hence, the flame-treated flat PDMS (FLT-F), PDMS-PIL (PIL-F), and PDMS-C-RESS (C-RESS-F) surfaces were fabricated. Note that the flame treatment process was not applied on the Teflon surface.

### 2.4. Surface Characterizations

A field-emission scanning electron microscope (FE-SEM, Hitachi S–4700, Hitachi High-technology Corporation, Tokyo, Japan) characterized the pattern qualities, including pattern shape, pattern size, and surface topology. An atomic force microscope (AFM, SPA400, Seiko Instruments Inc., Chiba, Japan) was used to measure the root-mean-square surface roughness (RMS roughness) of the PDMS samples. A contact angle goniometer (Ramé-hart Instrument Co., Model-400, Succasunna, NJ, USA) was used to characterize surface wettability and hydrophobicity. Droplets of 5.0 μL deionized water (DIW, γ_lv_ = 72.1 mN m^−1^) and ethylene glycol (EG, γ_lv_ = 47.7 mN m^−1^) were dropped onto the PDMS surfaces on five different regions across the samples. The tilt angle of the substrate was 0°. The measurement obtained an average water contact angle (WCA) and the ethylene glycol contact angle (EGCA).

### 2.5. Bacterial Anti-Adhesion Testing

#### 2.5.1. Bacterial Culture

Bacterial strain *Escherichia coli* (*E. coli*, NCTC 12923, bioMérieux, Sydney, Australia) was grown in tryptic soy broth (TSB) media (Difco Laboratories, Becton, Dickinson and Company, Sparks, MD, USA) at 37 °C by shaking at 200 rpm for 24 h. Then, 100 µL of *E. coli* cultured solution was spread on a tryptic soy agar (TSA) plate and incubated at 37 °C for 24 h. This working stock culture was kept at 4 °C.

#### 2.5.2. Crystal Violet Stain

Measurement of static biofilm formation followed the method from previous works [40,41] with some modifications. In this experiment, *E. coli* working stock was inoculated in TSB media by agitation at 37 °C for 24 h. The cell concentrations in the broth cultures were adjusted in the TSB media by measuring the absorbance in the range 0.01 to 0.02 arbitary units (a.u.), (λ = 540 nm) using an ultraviolet–visible (UV-VIS) spectrophotometer (Thermo Scientific Orion, AquaMate 8000, Göteborg, Sweden). The target concentration of *E. coli* cultures was approximately 10^6^ CFU/mL. Before testing, all PDMS samples (FLT, FLT-F, PIL, PIL-F, C-RESS, and C-RESS-F) and Teflon (control sample) were sterilized using an autoclave at 121 °C for 30 min with a saturated steam pressure of 15 pound force per square inch (psi).

Each sterilized sample was placed in 6-well plates that contained *E. coli* cultured in static conditions for 24 h. After that, the absorption at the wavelength of 570 nm was measured by the UV–VIS spectrometer to estimate the bacterial growth. Then, the samples were washed with DIW to remove loosely attached cells and were incubated with 95% ethanol (C_2_H_5_OH) for 3 min. After removing the 95% ethanol, the samples were incubated with 0.1% crystal violet for 30 min to stain the bacterial adhesion on the sample surfaces. After washing the samples with DIW, the staining cells were washed from the sample surfaces with acetic acid (33% conc.) by shaking at 150 rpm for 30 min. The absorption of the staining cell solution was measured at the wavelength of 591 nm to determine the bacterial adhesion on the sample surfaces [42]. Thus, the absorbance ratio of crystal violet stain and initial cell density was determined at the wavelengths of 591 and 570 nm (A591 nm/A570 nm). This absorbance ratio was used to compare the bacterial adhesion on different sample surfaces and determine the effectiveness of preventing biofilm formation. The bacterial adhesion on the sample surfaces was measured by FE-SEM (Hitachi S–4700, Hitachi High-technology Corporation, Tokyo, Japan).

## 3. Results

### 3.1. Surface Morphology and Surface Roughness

**Figure 5:**
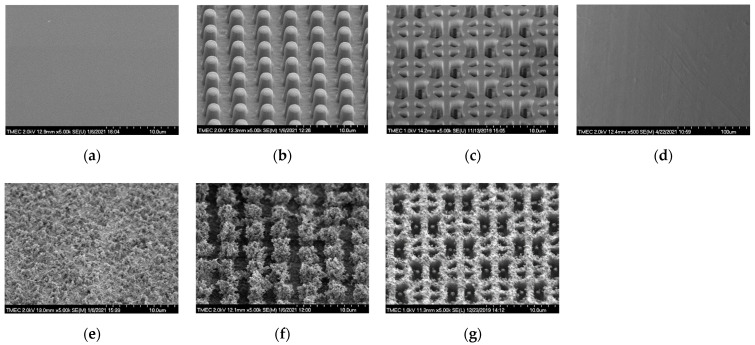
Side-view SEM images of untreated and flame-treated PDMS patterns and Teflon surfaces before bacterial adhesion testing: (**a**) FLT; (**b**) PIL; (**c**) C-RESS; (**d**) untreated Teflon; (**e**) FLT-F; (**f**) PIL-F; (**g**) C-RESS-F patterns.

**Figure 6:**
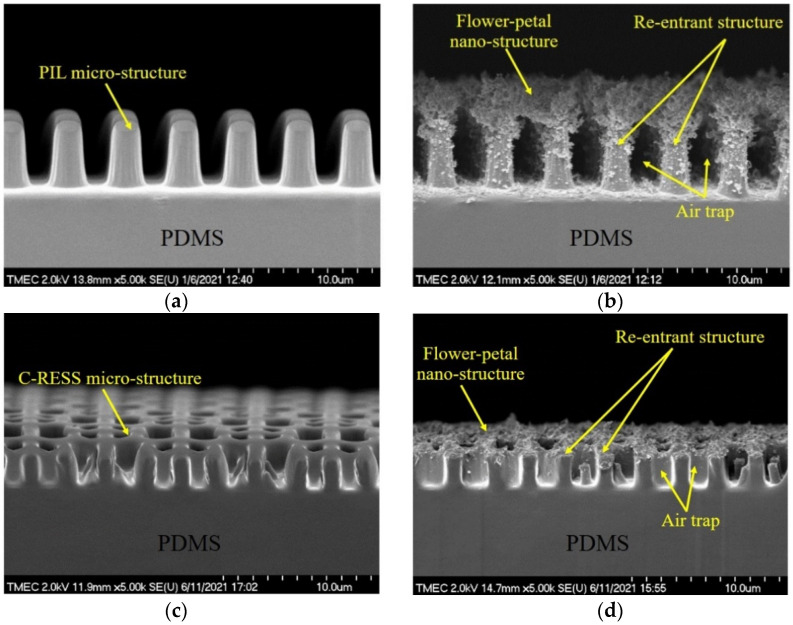
Cross-sectional SEM images of untreated and flame-treated PDMS patterns before bacterial adhesion testing: (**a**) PIL; (**b**) PIL-F; (**c**) C-RESS; (**d**) C-RESS-F patterns.

The side-view SEM images of the PDMS surfaces before and after flame treatment for 15 s are shown in Figure 5. Using HMDS priming, both PIL and C-RESS patterns were well replicated and successfully released from the Si master mold. Pattern mating, pattern clumping, and pattern collapsing were not found. Due to the limit of the resolution of the soft lithography process, nano-structures and nano-roughness did not develop on the PDMS surfaces, as shown in Figure 5a–c. After flame treatment for 15 s, wrinkles did not form on PDMS surfaces. This means the flame treatment process has a good uniform heating that does not induce surface thermal stress and micro-deformation [35]. However, flower petal nano-structures and nano-scale particles were formed on the flame-treated FLT-F, PIL-F, and C-RESS-F surfaces as shown in Figure 5e–g, respectively. The formation of the flower petal nano-structure on PDMS surfaces can be explained by the aggregation of the organosilicon compound in the PDMS matrix on the surface [35,38]. The cross-sectional SEM images in Figure 6a,c show that the pattern width (a) of the PIL pattern (a: 1.87 ± 0.3 μm) and C-RESS pattern (a: 0.50 ± 0.04 μm) were well-controlled within 10% deviation from the pattern design.

**Figure 7:**
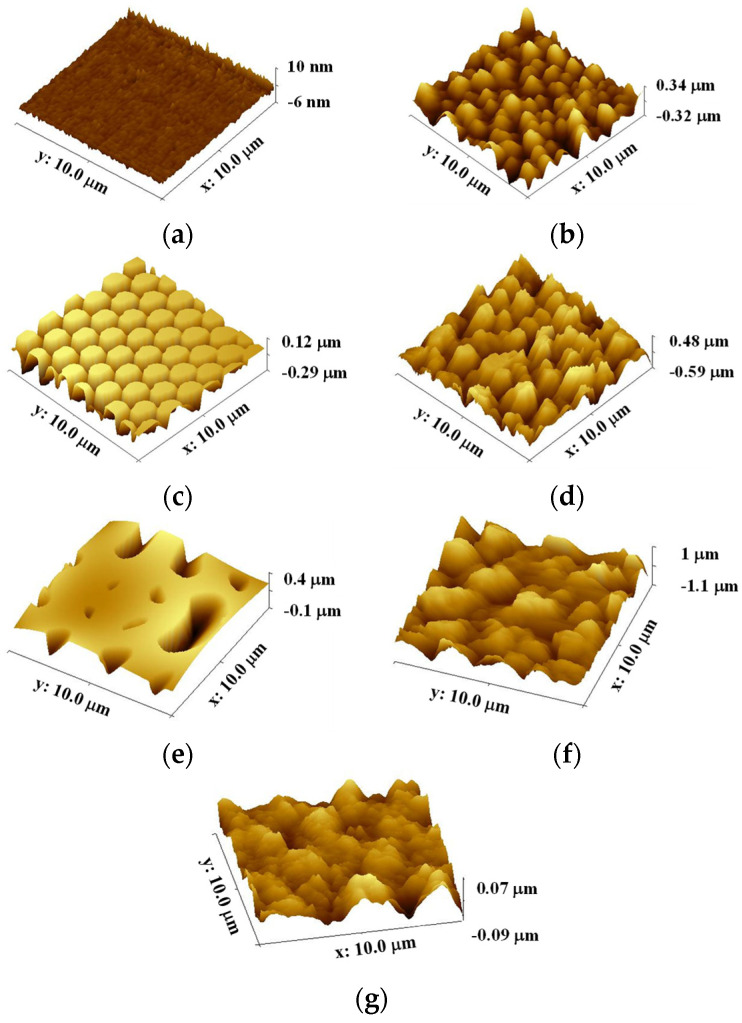
AFM images of untreated and flame-treated PDMS patterns and Teflon surfaces before bacterial adhesion testing: (**a**) FLT; (**b**) FLT-F; (**c**) PIL; (**d**) PIL-F; (**e**) C-RESS; (**f**) C-RESS-F; (**g**) untreated Teflon.

Both PIL and C-RESS patterns have smooth surfaces with nearly vertical sidewall profiles without sidewall scallop. The pattern heights (h) of PIL and C-RESS patterns were 4.62 ± 0.10 μm and 2.55 ± 0.07 μm, respectively. The variation in the Si etching rate on different densities of PIL and C-RESS micro-structures can be explained by the micro loading effect and the aspect ratio dependent etching (ARDE) effect [43,44]. Therefore, the average A.R.s of PIL and C-RESS micro-structures are approximately 2.47 and 5.10, respectively. After flame-treatment for 15 s, the pinch-off formation of flower petal nano-structures with a thickness of 3.14 μm generated voids (air gaps) between the adjacent PIL-F patterns by closing off the top surfaces. The flower petal nano-structures with the air gaps on the PIL-F pattern are similar to the re-entrant micro-structures [38]. In the case of the C-RESS-F pattern, the thickness of the flower petal nano-structures is around 1.0 µm. Therefore, the pattern heights (h) of the PIL-F and C-RESS-F patterns after the flame treatment process for 15 s were increased to 7.76 ± 0.13 μm and 3.55 ± 0.11 μm, respectively. This led to increases in the A.R.s of PIL-F and C-RESS-F micro-structures to 4.14 and 7.10, respectively. The different growth rates of flower petal nano-structures on the PIL-F and C-RESS-F patterns might be due to the different surface areas that make contact with the flame. The AFM results in Figure 7 show that the RMS roughness values of FLT, PIL, C-RESS patterns, and Teflon surfaces were 34.6, 92.6, 344.0, and 77.2 nm, respectively. After flame-treatment for 15 s, the RMS roughness values of the FLT-F, PIL-F, and C-RESS-F patterns were 129.6, 210.5, and 424.0 nm, respectively. The combination of the PIL micro-structure and flame treatment process can increase the RMS roughness of a flat PDMS surface from 34.6 to 210.5 nm.

### 3.2. Water and Ethylene Glycol Contact Angles

**Figure 8:**
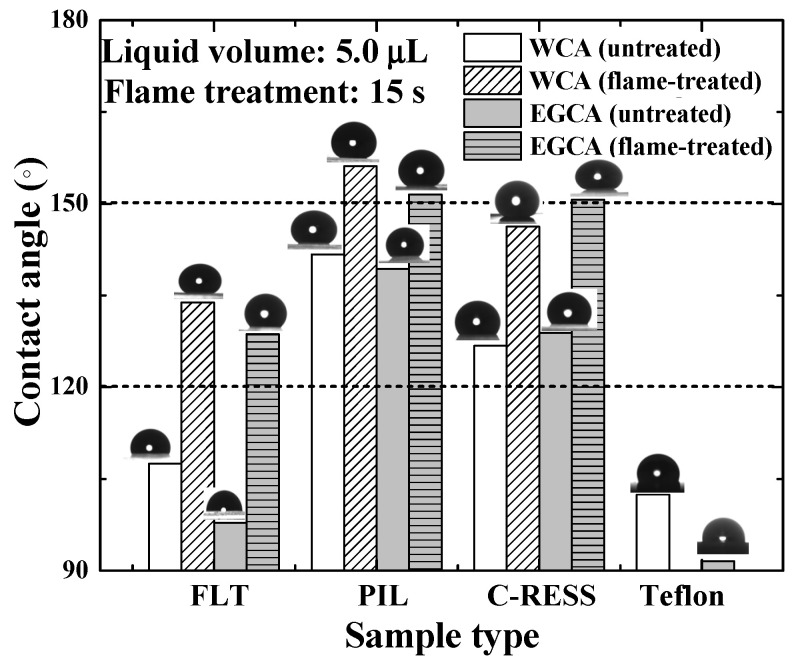
The effects of micro-structure geometry and the flame treatment process on the hydrophobic properties.

Figure 8 shows that the water contact angle (WCA) and ethylene glycol contact angle (EGCA) of the untreated FLT, PIL, C-RESS patterns, and Teflon surfaces were (107.5 ± 8.1°, 97.8 ± 7.3°), (141.7 ± 5.5°, 139.4 ± 2.3°), (126.8 ± 3.2°, 128.8 ± 5.0°), and (102.4 ± 5.1°, 91.5 ± 4.0°), respectively. After the flame treatment process for 15 s, the WCA and EGCA of FLT-F, PIL-F, and C-RESS-F patterns were (133.9 ± 3.8°, 128.6 ± 5.3°), (156.1 ± 1.5°, 151.5 ± 2.1°), and (146.3 ± 3.5°, 150.7 ± 1.8°), respectively. Due to the low surface roughness of flat PDMS and flat Teflon samples, the surface did not possess any superhydrophobic properties. After the flame treatment process, the PIL-F pattern showed superamphiphobic properties with the WCA and the EGCA greater than 150°. In contrast, the C-RESS-F pattern showed hydrophobic and superoleophobic properties. The summary of the measured pattern size, surface roughness, WCA, and EGCA of various PDMS patterns and Teflon surfaces before and after the flame treatment process for 15 s is shown in Table 1.

### 3.3. Static Biofilm Formation (Crystal Violet Stain) and Bacterial Anti-Adhesion

**Figure 9:**
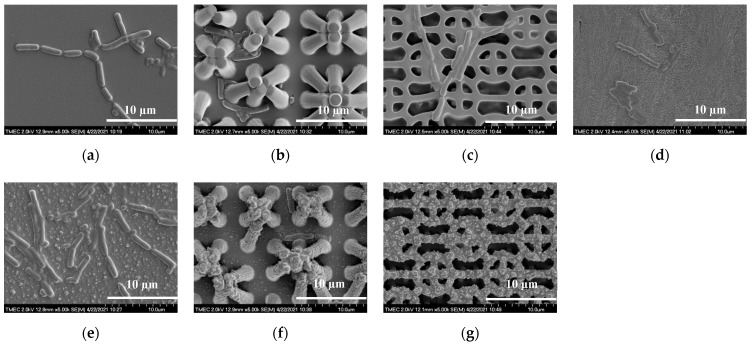
Top-view SEM images of PDMS and Teflon surfaces after bacterial anti-adhesion testing: (**a**) FLT; (**b**) PIL; (**c**) C-RESS; (**d**) untreated Teflon, (**e**) FLT-F; (**f**) PIL-F; (**g**) C-RESS-F.

Crystal violet (CV) staining was used to evaluate the effects of PDMS micro-structure and the flame treatment process on bacterial anti-adhesion and biofilm formation properties. Teflon was used as a control sample due to its low-surface-energy material (γ_s_ ≈ 19.6 mN m^−1^) with a hydrophobic property that is typically used to prevent bacterial adhesion on the surface. All PDMS and Teflon samples were incubated with a suspension of log-phase *E.coli* cells for 24 h for biofilm formation. Then, the samples were rinsed with DIW to remove the loosely adhered cells, and 95% ethanol was added to fix the attached cells, which were stained with CV. Figure 9a,d show that *E. coli* were attached on FLT and flat Teflon surfaces. This is related to poor hydrophobic properties on the surfaces. The flame-treated flat PDMS surface cannot prevent bacterial adhesion, as shown in Figure 9e. The pattern mating and clumping were observed on both PIL and PIL-F micro-patterns after bacterial anti-adhesion testing, as shown in Figure 9b,f, respectively. The pattern collapsing was caused by the poor mechanical strength of the PDMS and the geometry of the pillar micro-structure. Pattern mating and pattern clumping were caused by the van der Waals forces induced by the solutions used in the bacterial anti-adhesion testing. These weak electric forces between the adjacent patterns of both PIL and PIL-F micro-structures are stronger than pulling forces or recovery forces [45]. Moreover, the electrostatic discharge (ESD) generated during the bacterial anti-adhesion testing can induce an adhesion force between the adjacent PIL and PIL-F micro-structures. The severely damaged PIL and PIL-F patterns suppressed the hydrophobicity of the surface, resulting in the overall attachment of the *E. coli* on the spacing between pillar micro-structures (see Figure 9b,f).

However, a well-designed robust C-RESS micro-pattern has significantly reduced the severity of pattern damage from the applied external forces such as van der Waals forces and ESD [30]. Therefore, the C-RESS pattern can maintain the hydrophobicity and oleophobicity that led to the suppression of the bacterial adhesion, as shown in Figure 9c. In the case of the C-RESS pattern, *E. coli* adhered only on the top surface, which is entirely different from both PIL and PIL-F patterns. This might be due to the unique surface topology of the C-RESS pattern and its small pattern spacing, which reduced the contact area between the bacteria (length: 2.0 µm, diameter: 0.2–1.0 µm) and the C-RESS pattern (a: 0.5 µm, b: 0.5 µm). The attachment of *E. coli* on the C-RESS pattern was also related to its poor hydrophobicity (WCA: 126.8 ± 3.2°) and oleophobicity (EGCA: 128.8 ± 5.0°) properties. After flame treatment for 15 s, the C-RESS-F pattern with hydrophobicity (WCA: 146.3 ± 0.5°) and superoleophobicity (EGCA: 150.7 ± 1.8°) could show better bacterial anti-adhesion performance compared to other PDMS micro-structures, as shown in Figure 9g.

The attachment of the *E. coli* on the top surface and the pattern spacing of the C-RESS-F pattern were not observed. Therefore, the C-RESS and C-RESS-F patterns are considered attractive for bacterial anti-adhesion purposes. However, the surface roughness needs to be considered for bacterial adhesion on the material surfaces.

**Figure 10:**
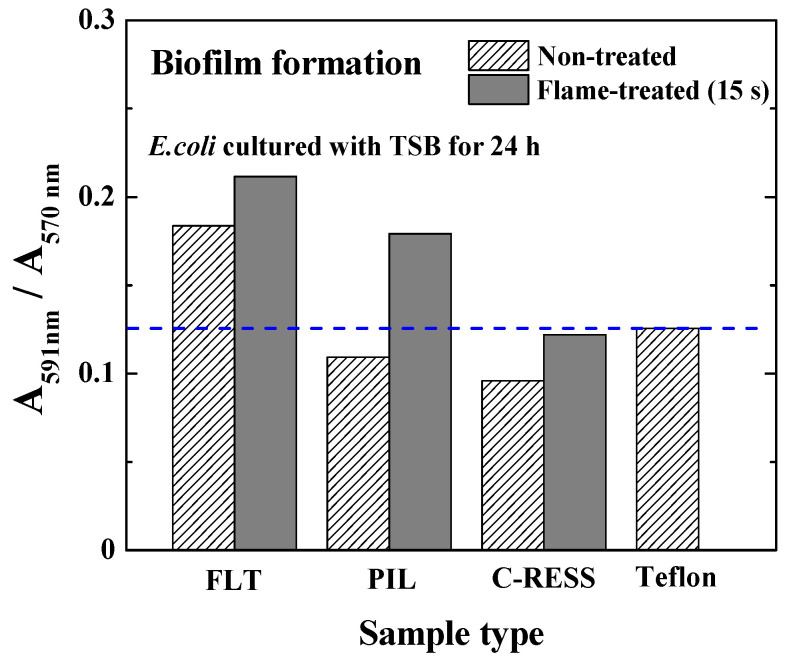
Micro-structures and flame treatment process affect the relative biofilm mass accumulation from the static biofilm formation per surface area (≈3.238 cm^2^). Note that the *E. coli* was cultured with TSB medium for 24 h.

As mentioned earlier, the absorbance ratios of the bacterial stain and bacterial culture per surface area (≈3.238 cm^2^) were used to determine the biofilm mass accumulation on the material surfaces. The results in Figure 10 show that the absorbance ratios (A591 nm/A570 nm) of FLT, PIL, C-RESS, and Teflon surfaces were 0.183, 0.109, 0.095, and 0.113, respectively. The bacterial adhesions on PIL and C-RESS patterns were 3.5%, and 15.9% reduced compared to the Teflon surface. Therefore, the unique surface topology of C-RESS and C-RESS-F patterns with a smaller size of micro-pattern (a: 0.5 µm, b: 0.5 µm) than the size of *E. coli* (a: 0.5 µm, length: 2.0 µm) can suppress the bacterial adhesion. After flame-treatment for 15 s, the absorbance ratio (A591 nm/A570 nm) of the C-RESS-F pattern was 42.6%, 31.8%, and 2.9% reduced compared to FLT-F, PIL-F, and Teflon surfaces, respectively. However, the bacterial anti-adhesion property of the C-RESS-F pattern is poorer than the C-RESS pattern but still comparable with Teflon.

## 4. Discussion

Based on the results, the flame-treated C-RESS micro-structure (C-RESS-F) exhibited hydrophobicity (WCA: 146.3 ± 0.5°) and superoleophobicity (EGCA: 150.7 ± 1.8°), which are considered attractive for bacterial anti-adhesion purposes because of three benefits. Firstly, the durability and the high A.R. (A.R.: 7.1) of the C-RESS-F pattern can maintain a decent level of surface roughness, hydrophobicity, and oleophobicity, reducing the adhesion strength of bacteria on the surface. Secondly, the formation of the flower petal re-entrant micro-/nano-structures on the C-RESS-F pattern during the flame treatment process might create air pockets in the pattern spacing. This air cushion generates a combination of solid–liquid and air–liquid interfaces on the C-RESS-F pattern [46,47]. Therefore, the contact area between the bacteria and the C-RESS-F pattern was reduced. Consequently, the interaction strength and surface adhesion force between the bacteria and the C-RESS-F pattern were also decreased, which led to the suppression of the biofilm formation on the surface [48]. Thirdly, the unique surface topology of C-RESS with a smaller size of micro-pattern (a: 0.5 µm, b: 0.5 µm) than the size of *E. coli* (a: 0.5 µm, length: 2.0 µm) can prevent the settlement of *E. coli* on the pattern spacing area and can also suppress the bacterial adhesion on the top surface. Therefore, the adhesion of *E. coli* on both C-RESS and C-RESS-F patterns is significantly reduced compared to other patterns with and without the flame treatment process, as shown in Figure 10.

**Figure 11:**
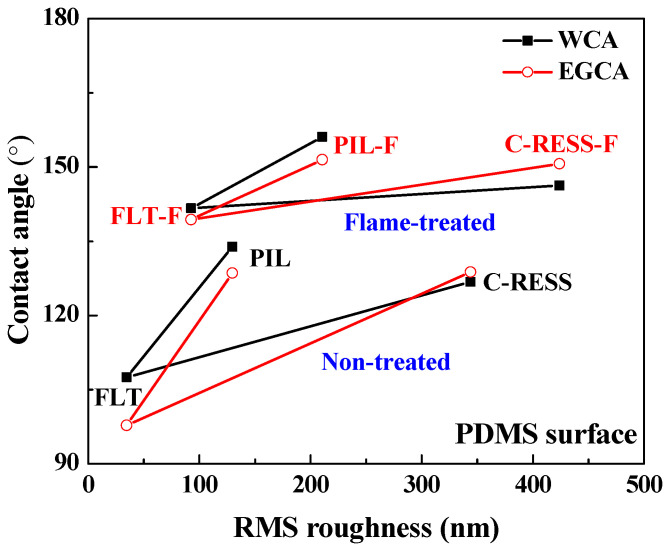
The effects of surface roughness of various PDMS patterns on water contact angle (WCA) and ethylene glycol contact angle (EGCA).

However, the flame treatment process increases the adhesion of *E. coli* on the FLT-F, PIL-F, and C-RESS-F surfaces. The geometry, including pattern shape, size, and surface roughness, might explain this phenomenon [49,50,51]. The results in Table 1 show that the pattern width (a) and pattern space (b) of the pillar and C-RESS patterns before and after the flame treatment process did not change. However, the surface roughness of FLT-F, PIL-F, and C-RESS-F patterns has increased compared to FLT, PIL, and C-RESS surfaces. This is due to the formation of the flower petal nano-structures during the flame treatment process by the aggregation of the organosilicon compound in the PDMS matrix on the surface [35,36,37,38]. The formation of flower petal nano-structures on PDMS surfaces led to increasing pattern height (h) and RMS surface roughness, as shown in Table 1. Figure 11 shows that when the surface roughness has increased, the WCA and EGCA have increased. The flame-treated PDMS samples have better hydrophobic and oleophobic properties than non-treated PDMS surfaces. Therefore, the flame treatment process should enhance the performance of bacterial anti-adhesion on the material surfaces [23,24,25].

**Figure 12:**
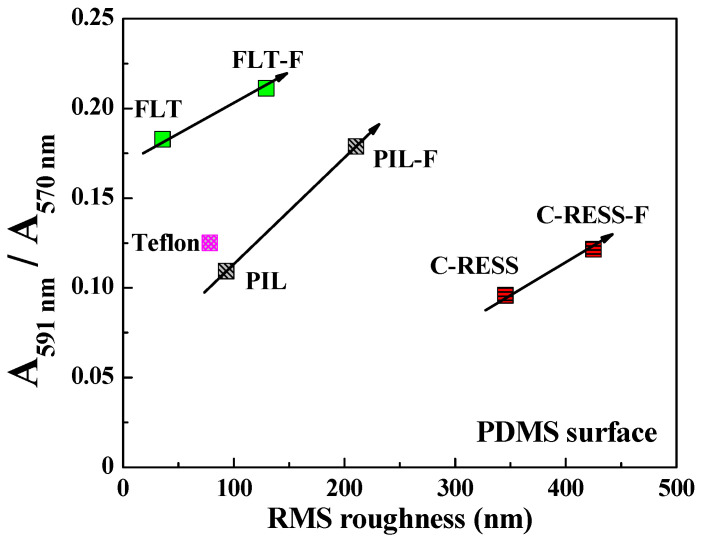
The effects of surface roughness of various PDMS patterns on the relative biofilm mass accumulation from the static biofilm formation per surface area (≈3.238 cm^2^). Note that the *E. coli* was cultured with TSB medium for 24 h.

However, high surface roughness increases the adhesion of *E. coli* on the flame-treated PDMS surfaces, as shown in Figure 12. This might be due to the excess flower petal nano-structures on the PDMS micro-structures that render more contact area for bacterial attachment. Hence, the rough surface on the flower petal nano-structure can induce more cell adhesion due to stimulating extracellular polymeric substances (EPSs) released from the cell, facilitating cell attachment and biofilm formation [52]. It was reported that *E. coli* preferentially chooses valleys between the pillar patterns to settle and form biofilms, even when the pattern size (a) is larger than the valleys (pattern spacing) [53]. In this experiment, the PIL pattern was designed with a width (a) of 2.0 μm and a pattern spacing (b) of 2.0 μm. The robust C-RESS pattern was designed with a width (a) of 0.5 μm and a pattern spacing (b) of 0.5 μm. After the flame treatment process, the flower petal nano-structure led to a smaller pattern spacing than untreated surfaces. Therefore, those PIL, C-RESS, PIL-F, and C-RESS-F pattern sizes are smaller than the size of *E. coli* (a: 0.5 µm, length: 2.0 µm), which can prevent the settlement of *E. coli* on the pattern spacing (valleys) area.

In Figure 9c,g, SEM images show that the *E. coli* adhered only on the top surface of the C-RESS-F pattern. However, the absorbance ratio (A591 nm/A570 nm) in Figure 10 reveals that the bacterial anti-adhesion property of the C-RESS-F pattern is poorer than the C-RESS pattern. This might be due to the excess nano-roughness on flower petal nano-structure increasing the surface area for bacterial attachment and providing a scaffold for adhesion [54,55]. It was reported that if the pattern height is larger than the bacteria size, the bacteria can easily adhere to the sidewall of the micro-structure to form the biofilm [49]. Therefore, the bacteria might have attached to the flower petal nano-structure and the sidewall area of the C-RESS-F micro-structure. Based on these results, the effect of surface roughness might overcome the superhydrophobic and superoleophobic properties on the bacterial anti-adhesion of the flame-treated PDMS patterns.

Not only the surface wettability, surface roughness, and surface topography, but the material’s surface charge density and stiffness are also effect the bacterial adhesion [54]. The surface charge on the bacterial adhesion is related to van der Waals forces and electrostatic interactions on the material surfaces. Typically, PDMS and Teflon have a negative charge on their surface [56,57]. Most bacteria also have a net negative surface charge due to carboxyl, amino, and phosphate groups on their cell wall surfaces. Therefore, the adhesion of bacteria is promoted on positively charged surfaces [58]. In this experiment, the bacterial adhesion on the flame-treated PDMS surface was greater than the untreated PDMS surface. This might be due to the flame treatment process inducing the positive charges on the PDMS micro-structure. The positive charges caused by the flame-treated PDMS surface were investigated by bacteria anti-adhesion testing using Gram-positive *Staphylococcus aureus* (*S. aureus*). The bacterial culture and measurement of static biofilm formation of *S. aureus* on the material surfaces were performed using a similar method to *E. coli*. Note that the shape and the size of *S. aureus* and *E. coli* are different.

**Figure 13:**
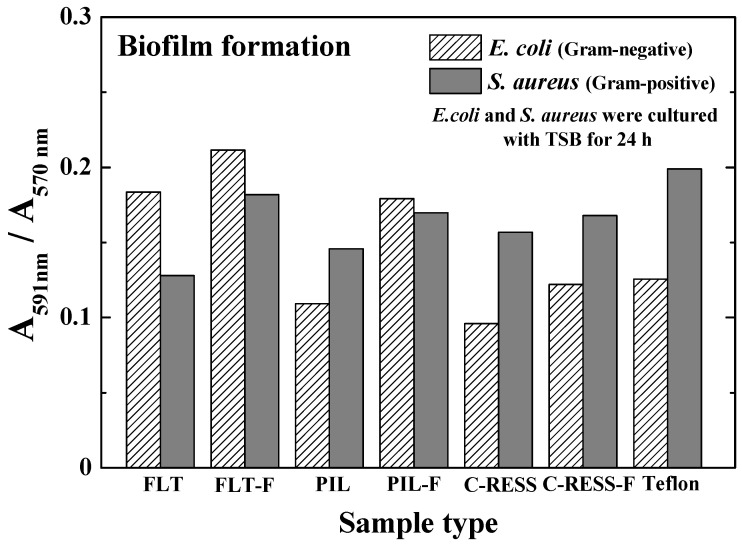
Comparison of the relative biofilm mass accumulation of *E. coli* (Gram-negative) and *S. aureus* (Gram-positive) from the static biofilm formation per surface area (≈3.238 cm^2^). Note that the *E. coli* and *S. aureus* were cultured with TSB medium for 24 h.

The results in Figure 13 show that Gram-positive *S. aureus* more easily adhered to the negative charge surface of Teflon and untreated PIL and C-RESS micro-structures than Gram-negative *E. coli*. Less adhesion of *S. aureus* on the FLT surface compared to *E. coli* might be due to the round shape of *S. aureus* with a smaller surface area (diameter: 0.5–1.0 μm) compared to the rod shape of *E. coli* (length: 2.0 μm, diameter: 0.2–1.0 μm). After flame-treatment for 15 s, Gram-negative *E. coli* more easily adhered to the FLT-F and PIL-F surfaces. This evidence supports that the positive charges might be induced on the flame-treated PDMS surface. In the case of the C-RESS-F micro-structure, *S. aureus* can easily adhere to the surface compared to *E. coli*. This might be due to the round shape of *S. aureus,* and its size is quite similar to the spacing of the C-RESS-F pattern. Therefore, *S. aureus* can easily adhere to the sidewall surface area and the top surface of the flower petal nano-structure compared to *E. coli*. Due to the shape and the size of *S. aureus*, which is smaller than the spacing of PIL, PIL-F, C-RESS, and C-RESS-F micro-structures, *S. aureus* can more easily adhere to the PDMS surfaces than *E. coli*.

Our previous study showed that the flame treatment could generate C–O bonds, resulting in the initial oxidation of the methyl groups during a combustion process [38]. It was reported that the oxide surface affects protein absorption and conformation to the surface, which influences an increase in the bacterial adhesion [59]. Hence, the oxide formation on the flower petal nano-structure can also increase the bacterial adhesion on flame-treated FLT-F, PIL-F, and C-RESS-F surfaces. However, an increase in the flame treatment duration resulted in suppressing C–O bonds on the PDMS surface [38]. Therefore, the condition of the flame treatment process to obtain the proper amount and size of flower petal nano-structure with well-controlled positive charges and oxide formation to improve the bacterial anti-adhesion property on the C-RESS-F pattern will be further investigated.

## 5. Conclusions

The simple flame treatment process is one of the alternative technologies to construct flower petal re-entrant micro-/nano-structures with a high surface roughness on the hierarchical PDMS micro-structures. After flame-treatment for 15 s, a well-designed robust C-RESS-F pattern showed hydrophobicity and superoleophobicity. The combination of durability and unique surface topology of the C-RESS-F pattern can prevent bacterial adhesion better than the conventional pillar micro-structure and is comparable with Teflon. The amount and size of the flower-petal nano-structure, the surface charges, and oxide formation after the flame treatment process are critical parameters for improving bacterial anti-adhesion properties. Therefore, the flame-treated C-RESS pattern (C-RESS-F) is one of the promising bacterial anti-adhesion micro-structures in practical utilization for medical applications and public areas.

## Figures and Tables

**Table 1:** Summary of the measured pattern size, surface roughness, WCA, and EGCA of various PDMS patterns and Teflon surfaces before and after flame treatment process for 15 s.

Sample Type	Pattern Size and Surface Roughness				Surface Wettability	
	Pattern Width (µm)	Pattern Height (µm)	A.R.	RMS Roughness (nm)	WCA (°)	EGCA (°)
FLT	NA	NA	NA	34.6	107.5 ± 8.1	97.8 ± 7.3
PIL	1.87 ± 0.30	4.62 ± 0.10	2.47	92.6	141.7 ± 5.5	139.4 ± 2.3
C-RESS	0.50 ± 0.04	2.55 ± 0.07	5.10	344.0	126.8 ± 3.2	128.8 ± 5.0
Teflon	NA	NA	NA	77.2	102.4 ± 5.1	91.5 ± 4.0
FLT-F	NA	NA	NA	129.6	133.9 ± 3.8	128.6 ± 5.3
PIL-F	1.87 ± 0.30	7.76 ± 0.13	4.14	210.5	156.1 ± 1.5	151.5 ± 2.1
C-RESS-F	0.50 ± 0.04	3.55 ± 0.11	7.10	424.0	146.3 ± 3.5	150.7 ± 1.8

## Data Availability

All results were analyzed by two independent observers. Data were measured from at least two different locations on the sample and presented as mean value ± standard deviation (SD). All data included in this study are available upon request by contact with the corresponding author.
